# The Relation of Dentistry to Medicine

**Published:** 1892-10

**Authors:** 


### THE RELATION OF DENTISTRY TO MEDICINE.

For a long time it has been a question of great interest whether dentistry is an independent profession, or whether it is a legitimate branch of medicine.

In times past many dental practitioners warmly declared that they were not specialists in medicine, because the medical conventions refused to recognize them as such. Some even declared that they would not be medical specialists if they could, as though individual preferences could make any difference in the relationship under discussion.

The wood-workers on a modern war-ship might refuse to recognize the iron-workers as ship-builders, but would clannish jealousy change the fact that they are all ship-builders?

From a general point of view, it might be said that, since the science of medicine embraces all pursuits which eliminate disease from the human body, dentistry must take a place as one of its legitimate branches.

But this statement might be considered a mere quibble, if it were not known that dentistry and general medicine must be intimately dependent upon each other if the best results are to be obtained.

On the welfare of the teeth depend a healthy digestion, and all pertaining thereto. A dental abscess or an aching nerve may profoundly disturb the nose, eye, ear, or throat. Anæmia and childbirth react seriously upon the health of the teeth; and the list might be almost indefinitely extended.

But perhaps some one may say, "Is not the origin of medicine and dentistry different? Did not dentistry spring from a race of fakirs and barbers, while medicine, from time immemorial, has been a profession of respectability and learning?

If this were so, would separate origins prove diverse professions? The Mississippi and the Missouri rise from different regions, but when they join does the diversity of their sources prevent them from forming a homogeneous, all-conquering stream?

Because barbers cupped, bled, and leeched, does that fact make those necessary operations any less professional or dignified in the eyes of the medical practitioner? Why, then, should the fact that barbers pulled teeth be so often quoted as a proof of dentistry's low origin? From this stand-point the barber would pose as an absolute connecting-link between general medicine and dentistry. But such an honor can hardly be accorded him.

From earliest days the care of the teeth has been an important part of medical education. In proof of this it might be well to quote a few lines from E. S. Talbot's opening address before the Section of Oral Surgery of the American Medical Association, June, 1891:

"Hippocrates speaks of dentifrices and fixing of teeth. Celsus, at the end of the first century, Ætius, in the sixth, Egenolff and Ambrose Paré in the fourteenth, all mention certain forms of treatment of the teeth. John Hunter, 1728, spent much time and study in dental treatment, as well as in the study of general anatomy. Fouchard, 1747, Bourdet, 1786, practised dentistry and wrote extensively on dental subjects. The instruments for dental as well as surgical purposes which are to be seen in the museums of Europe, together with the beautiful specimens of Etruscan and Phœnician dentistry now in possession of Drs. Van Marter of Rome, Barrett of Buffalo, and Taft of Cincinnati, are striking illustrations of the superior ability which men in early times acquired."

The last barrier was thrown down when dentistry was recognized by the American Medical Association as a branch of medicine. Each dentist is now tendered the inestimable advantage of personal contact with the medical profession at large. But does this mean that the schools of dentistry, as now organized, are to become obsolete, and that in time dentistry will be studied in medical colleges? Such an evolution is most improbable.

In reviewing the history of dentistry, it is noticeable that the men who have done the most to advance her interests have been without the title of M.D. This seems at first most remarkable, as the knowledge of general medicine is an unquestioned advantage; but, nevertheless, remarkable as this may appear, statistics prove it to be a fact.

The prominent men within the last fifty years who hold the simple title of D.D.S. first had their hands and eyes trained to perform the work of dentistry. For this reason, in later life, each medical fact was regarded from a dental point of view, and eagerly appropriated and modified to suit his operations. But the medical man with a dental education who has first had his interest aroused in all the specialties of medicine is apt to allow his attention to be diverted into three or four channels, and thus lacks the concentration and devotion which can alone bring success in a profession. A general practitioner who has dental training can hardly be expected to successfully compete with a dentist who has medical training; therefore, the independent college of dentistry, as it now stands, would seem an absolute necessity. The eye and hand must be trained long and carefully to perform their delicate work; the structures of the teeth must be impressed prominently on the student's mind, the artistic knowledge of facial restoration must be inculcated, and then let there be introduced into the course as much general medicine as may be expedient, but, so far as possible, let it be regarded from a dental stand-point. In a medical college such a course would not be feasible.

In closing, it may be said that while dentistry is a branch of medicine, it is such an extensive and important branch that its course of instruction must, of necessity, so differ from general medicine as to make them seem like separate professions.

